# Characteristics Predicting Short-Term and Long-Term Health-Related Quality of Life in Patients with Esophageal Cancer After Neoadjuvant Chemoradiotherapy and Esophagectomy

**DOI:** 10.1245/s10434-023-14028-8

**Published:** 2023-08-16

**Authors:** Berend J. van der Wilk, Ben M. Eyck, Bo J. Noordman, Leonieke W. Kranenburg, Mark Oppe, Sjoerd M. Lagarde, Bas P. L. Wijnhoven, Jan J. Busschbach, J. Jan B. van Lanschot

**Affiliations:** 1 Department of Surgery, Erasmus MC Cancer Institute, University Medical Center, Rotterdam, The Netherlands; 2 Department of Psychiatry, Section of Medical Psychology and Psychotherapy, Erasmus MC–University Medical Center, Rotterdam, The Netherlands; 3 Maths in Health, Rotterdam, The Netherlands

**Keywords:** Esophageal cancer, Neoadjuvant chemoradiotherapy, Health-related quality of life, Active surveillance

## Abstract

**Background:**

Esophagectomy is associated with lasting effect on health-related quality of life (HRQOL). Patients desire detailed information on the expected impact of treatment on their postoperative HRQOL. The aim of the present study is to identify clinicopathological characteristics predictive for changes in short-term and long-term HRQOL after neoadjuvant chemoradiotherapy (nCRT) and surgery.

**Methods:**

HRQOL was measured using EORTC-QLQ-C30 and QLQ-OES24 questionnaires prior to nCRT, three, six, nine and twelve months postoperatively and at a minimum of six years postoperatively. Based on previous experience and available literature, several subgroups were predefined for different clinicopathological characteristics: baseline global HRQOL, WHO performance status, histology, tumor stage and tumor location. The primary endpoints of the present study were the change compared to baseline in the HRQOL dimensions physical functioning and eating problems. Secondary endpoints were global HRQOL, fatigue and emotional problems.

**Results:**

In total, 134 (76%) of 177 patients who received HRQOL questionnaires, responded at baseline. Patients who reported a high baseline global HRQOL had a more severe deterioration in eating problems (+14.5 to + 18.0), global HRQOL (-16.0 to -28.0) and fatigue (+10.5 to +14.9) up to six years postoperatively compared to patients who reported a low baseline global HRQOL. Patients who had stage 2 tumor (UICC 6th edition) had a more severe deterioration in eating problems (+14.6 to +19.0) and global HRQOL (-10.1 to -17.1) than patients who had stage 3 tumor.

**Conclusions:**

The results suggest that patients with locally advanced esophageal cancer in favorable condition at baseline decline more in terms of various HRQOL outcomes.

**Supplementary Information:**

The online version contains supplementary material available at 10.1245/s10434-023-14028-8.

Neoadjuvant chemoradiotherapy followed by esophagectomy is a standard treatment for locally advanced esophageal cancer.^1–3^ This treatment is associated with severe postoperative complications, lasting symptoms and a detrimental effect on health-related quality of life (HRQOL).^4–6^ HRQOL is increasingly considered an important outcome measure in clinical trials. Moreover, patients report that they desire detailed information about the impact of esophagectomy on their postoperative outcomes, including HRQOL.^7^ Postoperative HRQOL is considered one of the most important factors for patients to be informed about in the surgical management of esophageal cancer.^8^ Therefore, clinicians should have knowledge of the expected postoperative HRQOL after neoadjuvant chemoradiotherapy and esophagectomy, and discuss this with their patients prior to final treatment decision.

Although surgery itself is associated with a decrease in postoperative HRQOL, the extent of surgery (e.g*.*, extended transthoracic versus transhiatal) did not influence the postoperative HRQOL course.^9–12^ In addition, the addition of neoadjuvant chemoradiotherapy did not influence HRQOL postoperatively.^13,14^ Clinical or pathological factors prior to initiation of treatment, however, can influence postoperative HRQOL.^15^ For instance, histology of the tumor, the presence of comorbidities, the location of the tumor, and tumor stage are predictive for worse HRQOL 6 months after primary esophagectomy.^15^ In addition, preoperative HRQOL is prognostic for long-term survival and for the risk of postoperative complications, possibly resulting in a deterioration of postoperative HRQOL.^16–19^

Studies thus far have focused on predictive factors for worse HRQOL in patients mainly undergoing primary esophagectomy, with follow-up rarely beyond 6 months postoperatively. The aim of the present study was to identify characteristics predictive for changes in short-term HRQOL (up to 12 months postoperatively) and long-term HRQOL (more than 6 years postoperatively) in patients who underwent neoadjuvant chemoradiotherapy followed by esophagectomy. For this purpose, we compared deterioration in postoperative HRQOL course for several subgroups.

## Patients and Methods

### Sources of Data

The randomized ChemoRadiotherapy for Oesophageal cancer followed by Surgery Study (CROSS)-trial was conducted between 2004 and 2008. In this trial, neoadjuvant chemoradiotherapy followed by surgery was compared with surgery alone in patients with locally advanced resectable adenocarcinoma or squamous cell carcinoma of the esophagus or esophagogastric junction. Results of this trial have already been reported.^1,2,20^ During the trial, HRQOL questionnaires have been provided at several time points to assess the influence of neoadjuvant chemoradiotherapy on postoperative HRQOL. Both the short-term and the long-term effects of neoadjuvant chemoradiotherapy on postoperative HRQOL have been published previously.^13,14^ For the current study, the HRQOL data of patients who underwent neoadjuvant chemoradiotherapy followed by surgery within the CROSS-trial were used.

### HRQOL Questionnaires

Questionnaires were available that had been provided to measure short-term HRQOL, i.e., prior to treatment (baseline), 3 months, 6 months, 9 months, and 12 months postoperatively and to measure long-term HRQOL, i.e., at a minimum follow-up of 6 years postoperatively. Cancer-specific HRQOL had been measured with the European Organisation for Research and Treatment of Cancer (EORTC) Quality of Life Questionnaire – Core 30 (QLQ-C30). This questionnaire consists of five functional scales (physical functioning, role functioning, emotional functioning, cognitive functioning, and social functioning), three symptom scales (fatigue, nausea/vomiting, and pain) and one global HRQOL scale. Esophageal-cancer-specific HRQOL had been measured with the EORTC QLQ-Oesophageal Cancer Module 24 (QLQ-OES24). This questionnaire consists of six symptom scales (dysphagia, deglutition problems, eating problems, gastrointestinal symptoms, pain, and emotional problems) and four single symptom items (dry mouth, troublesome coughing, troublesome talking, hair loss). The scales have four response subcategories: (1) not at all, (2) a little, (3) quite a bit, and (4) very much, according to a Likert scale. The global HRQOL has a 7-point scale ranging from poor to excellent. A higher score on a functional scale represents a better outcome for patients in that domain, while a higher score on a symptom scale represents a worse outcome for patients in that domain.

Endpoints were previously defined by consensus discussion with experienced medical oncologists, upper GI surgical oncologists, and a nurse practitioner. Endpoints were based on literature, clinical relevance, and hypothesized association with short- and long-term outcomes of neoadjuvant chemoradiotherapy (nCRT) plus esophagectomy.^13–15,19,21,22^ The primary endpoints of this study were physical functioning (QLQ-C30) and eating problems (QLQ-OES24), whereas secondary outcomes were global HRQOL (QLQ-C30), fatigue (QLQ-C30), and emotional problems (QLQ-OES24).

### Patient and Tumor Characteristics

Several factors that have been described in the literature to potentially influence postoperative HRQOL are age, gender, extent of surgery, addition of nCRT, comorbidities, tumor stage, tumor location, and histology.^9,13–17,21,23,24^ To avoid overfitting of our model and to prevent the risk of obtaining statistically significant results on the sole basis of multiple testing errors, we deliberately limited the number of clinicopathological factors to five, which could be determined prior to treatment and were chosen prior to start of analyses. On the basis of clinical experience and literature, these selected factors were: baseline global HRQOL, World Health Organization (WHO) performance status, histology, tumor stage, and tumor location.^15–18^ These variables were categorized and accordingly, patients were divided into subgroups:

- Low versus high baseline global HRQOL (defined as below or above the median EORTC score of 75 on the global HRQOL domain of the EORTC-QLQ-C30 questionnaire prior to treatment);
- WHO-0 (fully active) versus WHO-1 (restricted in strenuous physical activity) performance status (patients with ≥ WHO-2 were not included in the CROSS-trial);
- Tumor histology: adenocarcinoma versus squamous cell carcinoma;
- Tumor stage 2 versus stage 3 [according to the 6th Union for International Cancer Control (UICC) tumor, node, metastasis (TNM) classification^25^];
- Tumor located in the proximal or middle third of the esophagus versus tumors in the distal third of the esophagus versus tumors at the esophagogastric junction (as determined by endoscopy).

**Fig. 1:**
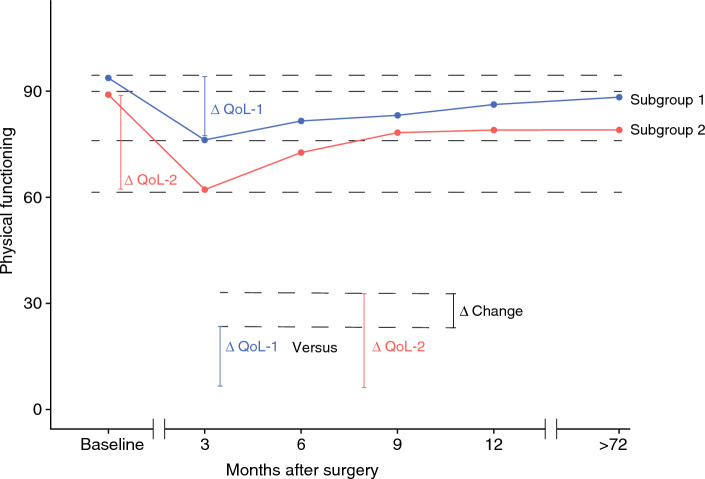
In this explanatory figure it is visualized how the impact of a specific baseline factor (e.g. histology: subgroup 1 is adenocarcinoma, subgroup 2 is squamous cell carcinoma), on a specific domain of HRQOL (e.g. physical functioning) was quantified. The change in HRQOL in relation to baseline HRQOL is expressed as ΔQoL in each of the two subgroups (ΔQoL-1 versus ΔQoL-2) at several postoperative timepoints. The difference in change between the two subgroups at a specific timepoint (in this example: three months postoperatively) is expressed as Δ Change. In a functional scale (e.g. physical functioning), a positive Δ Change represents improved recovery for patients compared to the reference subgroup. In a symptom scale (e.g. eating problems) a positive Δ Change represents worse recovery compared to the reference subgroup

Between the two subgroups of each clinicopathological factor, the postoperative course of HRQOL was compared. This is further explained in Fig. 1.

### Statistical Analysis

Questionnaire scores were computed according to the EORTC scoring manual. Effects over time were assessed with multilevel mixed model analysis. This method can effectively handle incomplete cases over time.^26^ Patients formed the upper level, whereas repeated HRQOL measures of the patients formed the lower level of the multilevel regression model. For each (primary and secondary) outcome a separate model was postulated. The differences between the baseline scores and the scores at each point in time (i.e., the gradients compared to baseline) were the dependent variables in the models. This was preferred over the scores at each point in time because the change in HRQOL was the focus of this study. STATA mixed command maximum likelihood estimation with the robust option for estimating the variance was used for all models. Both random slope and random intercept model specifications were tested. In the final models, time and the predefined covariates for which we adjusted in the models (i.e., age, gender, WHO performance status, histology, tumor stage, and tumor location) were considered. In addition to the statistical analyses, the clinical relevance of the differences was assessed as well. A difference of more than 10 EORTC points was considered a clinically relevant difference. A difference of 5–10 points was considered a minimally relevant difference, a difference of 10–15 points was considered a moderately relevant difference, and a difference of > 15 points was considered a highly relevant difference.^28^ Per outcome, five comparisons were done (3 months, 6 months, 9 months, 12 months, and > 6 years postoperatively). To correct for multiple testing, a Bonferroni correction was applied, resulting in a statistical significance level of 0.05/5 = 0.01. Hence, *p* < 0.01 was considered statistically significant.

## Results

### Patients and Response Rate

A total of 177 of 180 patients who were assigned to the neoadjuvant chemoradiotherapy plus surgery arm received baseline HRQOL questionnaires. Baseline characteristics of all 177 patients are summarized in Table 1. Of the eligible patients, 69–94% responded and returned the questionnaire at various time points. A detailed overview of the response rates is presented in Table 2.

**Table 1:** Baseline characteristics of eligible patients

	Total: 177	%
Age (years)		
Median	60	
IQR	55–67	
Gender		
Male	134	75
Female	43	25
WHO performance status*		
0	144	81
1	33	19
Histology		
Adenocarcinoma	134	75
Squamous cell carcinoma	40	23
Other	3	2
cT		
1	1	< 1
2	26	15
3	149	84
Unknown	1	< 1
cN		
0	59	33
1	115	65
Unknown	3	2
Tumor stage**		
2	73	41
3	101	57
Unknown	3	2
Tumor location^$^		
Proximal third	4	2
Middle third	24	14
Distal third	104	58
GEJ	39	22
Unknown	6	3

*WHO-0 indicates fully active patients and WHO-1 indicates patients who are unable to carry out heavy physical work

**Classified according to the 6th edition of the Union for International Cancer Control’s (UICC) TNM staging manual

^$^Tumor location determined with endoscopy

*cN* clinical nodal stage, *cT* clinical tumor stage, *GEJ* gastroesophageal junction, *IQR* interquartile range, *WHO* World Health Organization

**Table 2:** Detailed overview of number of returned (%) questionnaires per subgroup

Postoperative timepoint (months)	Baseline	3	6	9	12	> 72
Eligible	177 (100)	163 (100)	151 (100)	145 (100)	136 (100)	70 (100)
Returned in total (%)	134 (76)	119 (73)	113 (75)	103 (71)	94 (69)	66 (94)
Baseline gHRQOL						
Low	50 (28)	34 (21)	32 (12)	26 (18)	24 (18)	17 (24)
High	84 (47)	58 (36)	56 (37)	54 (37)	52 (38)	37 (53)
No baseline HRQOL available	0 (0)	27 (17)	25 (17)	23 (16)	18 (13)	12 (17)
WHO performance status						
0	110 (62)	97 (60)	94 (62)	86 (59)	79 (58)	54 (77)
1	24 (14)	22 (13)	19 (13)	17 (12)	15 (11)	12 (17)
Histology						
Adenocarcinoma	100 (56)	89 (55)	84 (56)	77 (53)	69 (51)	48 (69)
Squamous cell carcinoma	31 (18)	28 (17)	26 (17)	24 (17)	22 (16)	18 (26)
Other	3 (2)	2 (1)	3 (2)	2 (1)	3 (2)	0 (0)
Tumor stage						
2	58 (33)	54 (33)	48 (32)	48 (33)	45 (33)	34 (48)
3	73 (41)	63 (39)	63 (42)	54 (37)	47 (35)	31 (44)
Unknown	3 (2)	2 (1)	2 (1)	1 (1)	2 (1)	1 (1)
Tumor location						
Proximal third	4 (2)	4 (2)	4 (3)	3 (2)	2 (1)	0 (0)
Middle third	16 (9)	15 (9)	13 (9)	10 (7)	8 (6)	9 (13)
Distal third	80 (45)	71 (44)	66 (44)	60 (41)	57 (42)	34 (49)
GEJ	28 (16)	25 (15)	27 (18)	26 (18)	22 (16)	18 (26)
Unknown	6 (3)	4 (2)	3 (2)	4 (3)	5 (4)	5 (7)

*GEJ* Gastroesophageal junction, *gHRQOL* Global health-related quality of life, *WHO* World Health Organization

### Primary Endpoints

Physical Functioning and Eating Problems

**Fig. 2:**
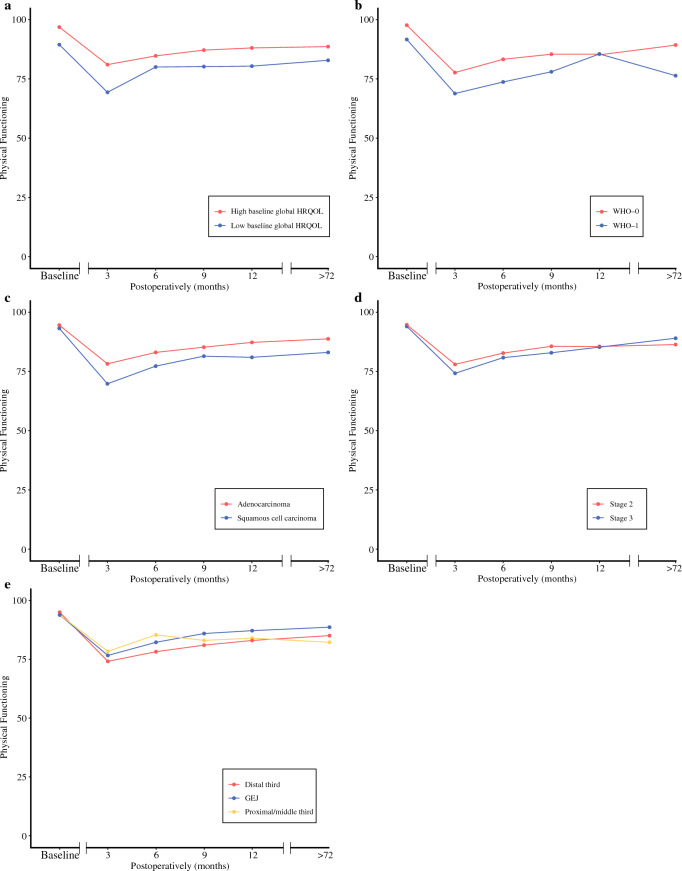
Mean EORTC-scores representing the physical functioning for patients with **a** high baseline global HRQOL (red line) or low baseline global HRQOL (blue line) **b** WHO-0 (red line) or WHO-1 (blue line) **c** adenocarcinoma (red line) or squamous cell carcinoma (blue line) **d** stage 2 tumors (red line) or stage 3 tumors (blue line) **e** tumors located in the proximal/middle third (orange line), tumors in distal third of the esophagus (red line), or tumors at the GEJ (blue line). No significant differences in deterioration of physical functioning between subgroups were seen (see also Table 3)

**Fig. 3:**
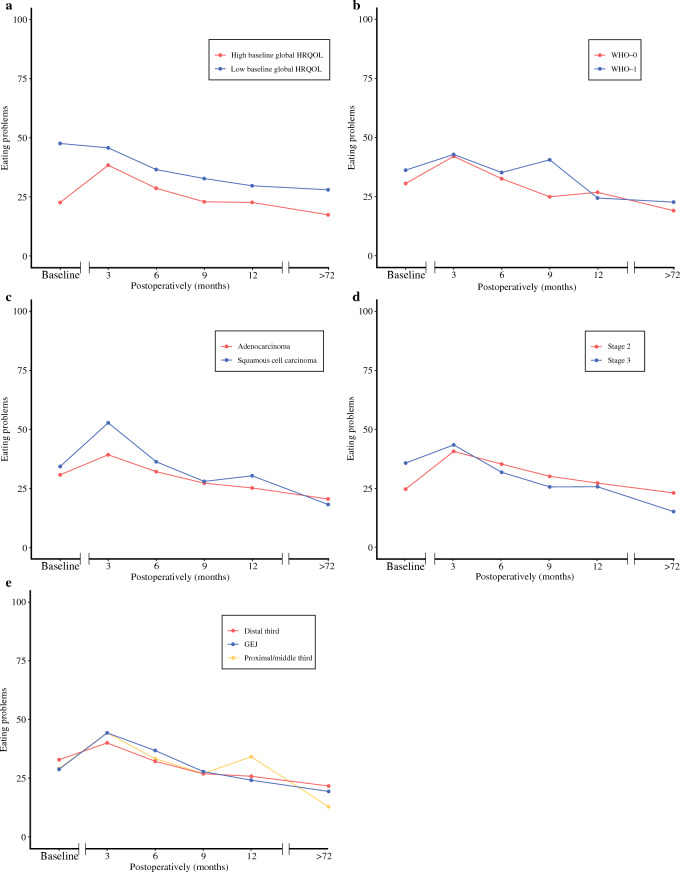
Mean EORTC-scores representing the eating problems for patients with **a** high baseline global HRQOL (red line) or low baseline global HRQOL (blue line) **b** WHO-0 (red line) or WHO-1 (blue line) **c** adenocarcinoma (red line) or squamous cell carcinoma (blue line) **d** stage 2 tumors (red line) or stage 3 tumors (blue line) **e** tumors located in the proximal/middle third (orange line), tumors in distal third of the esophagus (red line), or tumors at the GEJ (blue line). Patients with high baseline global HRQOL had a clinically relevant, more severe deterioration in short-term and long-term eating problems compared to patients with low baseline global HRQOL. The same is true for patients with stage 2 tumor compared to patients with stage 3 tumor. See also Table 4

HRQOL curves for physical functioning and eating problems of all five subgroups are reported in Figs. 2 and 3, respectively. No significant differences in deterioration of physical functioning between subgroups were seen (Table 3). The group of patients with a high baseline global HRQOL had a clinically relevant, more severe deterioration and/or worse recovery in both short-term and long-term eating problems. Differences in change compared with baseline for all time points ranged from + 14.5 to + 18.0 points, as summarized in Table 4. Patients with a stage 2 tumor had a clinically relevant, more severe deterioration in short-term and long-term eating problems compared with patients with stage 3 tumor. Differences in change compared with baseline from 6 months to > 72 months postoperatively ranged from + 12.6 to + 19.0. Differences in deterioration of eating problems between subgroups are summarized in Table 4.

**Table 3:** Overview of differences in improvement or deterioration with respect to physical functioning between subgroups

	Postoperative time point (months)									
	3		6		9		12		> 72	
	Δ	p	Δ	p	Δ	p	Δ	p	Δ	p
Baseline gHRQOL										
Low* versus high	4.2	0.85	−2.7	0.15	−0.5	0.14	0.2	0.38	−1.7	0.68
WHO										
0* versus 1	−5.8	0.13	−6.5	0.25	−4.5	0.30	3.1	0.52	−10.0	0.05
Histology										
AC* versus SCC	7.0	0.85	−4.4	0.80	−2.5	0.66	−4.9	0.56	−4.4	0.80
Tumor stage										
2* versus 3	−3.0	0.19	−1.2	0.59	−2.0	0.26	0.4	0.94	3.4	0.09
Tumor location										
Distal* versus prox/mid	−1.6	0.11	−3.0	0.27	3.1	0.87	3.4	0.35	6.6	0.80
GEJ* versus prox/mid	−5.3	0.05	−8.2	0.12	−3.1	0.38	−2.0	0.19	1.9	0.87
GEJ* versus distal	−3.7	0.33	−5.2	0.40	−6.1	0.32	−5.3	0.42	−4.8	0.60

Δ Difference between subgroups in improvement or deterioration compared with baseline; a negative value represents a deterioration and a positive value represents an improvement with respect to physical functioning compared with the reference subgroup

*Reference subgroup

*AC* adenocarcinoma, *GEJ* gastroespohageal junction, *gHRQOL* global health related quality of life, *SCC* squamous cell carcinoma, *WHO* World Health Organization performance status

**Table 4:** Overview of differences in improvement or deterioration with respect to eating problems between subgroups

	Postoperative timepoint (months)									
	3		6		9		12		> 72	
	Δ	*p*	Δ	*p*	Δ	*p*	Δ	*p*	Δ	*p*
Baseline gHRQOL										
Low* versus high	17.6	0.08	17.1	0.02	15.2	0.20	18.0	0.11	14.5	0.08
WHO										
0* versus 1	−14.9	0.33	−3.1	0.12	10.0	0.78	−8.1	0.27	−2.1	0.31
Histology										
AC* versus SCC	10.0	0.90	0.6	0.42	−2.8	0.75	1.7	0.97	−5.9	0.13
Tumor stage										
2* versus 3	−8.4	0.89	−14.6	0.31	−15.6	0.53	−12.6	0.31	−19.0	0.12
Tumor location										
Distal* versus prox/mid	−7.9	0.41	−4.8	0.24	−3.9	0.07	−12.0	0.08	5.1	0.83
GEJ* versus prox/mid	0.4	0.85	3.9	0.75	1.1	0.32	−9.6	0.17	6.9	0.93
GEJ* versus distal	8.3	0.26	8.7	0.40	5.0	0.37	2.4	0.93	1.8	0.92

Δ Difference between subgroups in improvement or deterioration compared to baseline; a negative value represents an improvement and a positive value represents a deterioration with respect to eating problems compared to the reference subgroup

*Reference subgroup

*AC* adenocarcinoma, *GEJ* gastroesophageal junction, *gHRQOL* global Health Related Quality Of Life, *SCC* squamous cell carcinoma, *WHO* World Health Organization performance status

### Secondary Endpoints

#### Global HRQOL

HRQOL curves for global HRQOL of all subgroups are reported in Supplementary Fig. 1. Compared with patients with low baseline global HRQOL, the group of patients with a high baseline global HRQOL had clinically relevant and statistically significant more severe deterioration in short- and long-term global HRQOL postoperatively (from –16 to –28). Patients with a stage 2 tumor had clinically relevant more severe deterioration and/or worse recovery in short-term and long-term global HRQOL postoperatively than patients with a stage 3 tumor (from − 16.6 to − 17.1). Differences in deterioration of global HRQOL between subgroups are summarized in Supplementary Table 1.

#### Fatigue and Emotional Problems

HRQOL curves for fatigue and emotional problems of all subgroups are reported in Supplementary Figs. 2 and 3, respectively. Patients with high baseline global-health-related quality of life had a clinically relevant, more severe deterioration in fatigue compared with patients with low global-health-related quality of life (+ 10.5 to + 14.9). No statistically significant differences were seen between subgroups in emotional problems. Differences in deterioration of fatigue and emotional problems between subgroups are summarized in Supplementary Tables 2 and 3, respectively.

## Discussion

Patients with high baseline global HRQOL had a more severe deterioration and/or worse recovery in short-term and long-term postoperative eating problems, global HRQOL, and fatigue compared with patients with low baseline global HRQOL. Patients with stage 2 tumor had a clinically relevant, more severe deterioration in eating problems and global HRQOL compared with patients with a stage 3 tumor.

An earlier study by Djarv et al. reported on predictors of postoperative quality of life 6 months after primary esophagectomy.^15^ In that study, a cross-sectional analysis was performed showing that patients with at least one comorbidity, patients with squamous cell carcinoma, and patients with a tumor in the middle or upper part of the esophagus had a higher risk for poor HRQOL at 6 months postoperatively. It is expected that patients with unfavorable background variables (e.g., having comorbidities) report lower HRQOL. In the present study, the influence of nCRT followed by surgery on deterioration of physical functioning, eating problems, fatigue, global HRQOL, and emotional problems was compared between subgroups, as explained in explanatory Fig. 1. This does not simply mean, however, that patients who had a more severe deterioration in a specific HRQOL domain also had worse absolute EORTC scores on that specific domain, compared with other patients. For example, patients with a high baseline global HRQOL had more severe deterioration in eating problems than patients with a low baseline global HRQOL. The EORTC scores postoperatively, however, did not differ substantially. Although we have not formally compared these scores, differences did not exceed 10 points in absolute EORTC scores (data not shown), which was the threshold in the present study for a clinically relevant difference.^27^ It remains a topic of discussion whether the deterioration or the absolute postoperative HRQOL scores are more relevant for individual patients and should be compared with each other.^28^ What the present longitudinal study adds to the earlier study by Djarv et al. is that the decline in HRQOL outcomes seem to be more prominent in patients with favorable conditions at baseline. Or, stated differently, patients with favorable conditions seem to benefit less from treatment compared with their starting conditions in terms of health-related quality of life.

We believe that the greater drop in HRQOL for patients with a good baseline HRQOL and with stage 2 tumors is at least partly reflected by the patients’ expectations. In measuring health-related quality of life, we do not take into consideration the way patients arrive at these judgements. A model described earlier by Carr et al. stated that health-related quality of life is the gap between our expectations of health and our experience of it: a senior person who can still walk to the bus stop will consider his mobility satisfying, while a younger person will consider “only able to walk” as minimal mobility.^29^ Possibly, patients with lower baseline global HRQOL and patients with higher staged (i.e., stage 3) tumors already have a lower expectation of the HRQOL to come after the treatment. In this sense, the gap between the expectations and their experience will be smaller than when a patient starts with a relatively good HRQOL. Furthermore, patients with higher staged tumors could experience more symptoms already at baseline (e.g., dysphagia), which might partly dissolve during neoadjuvant chemoradiotherapy. The adverse effects of neoadjuvant therapy could be perceived as mild because of the trade-off for less tumor-related symptoms.

The willingness of surgeons to operate on patients with a good condition seems higher compared with patients with poor condition, which is reflected by trial inclusion criteria and clinical guidelines.^1,30^ This could be explained by the supposed impact and relatively high mortality of an esophagectomy. Our results show that it is especially this group of patients with a good condition (reflected by high baseline global HRQOL and low staged tumors) who show the most severe deterioration in HRQOL after esophagectomy. Therefore, the expectations of these patients should be especially managed by providing information about the impact of nCRT and surgery on their HRQOL, as they have more to lose in this respect.

Most probably, the deterioration in HRQOL postoperatively can be ascribed to the impact of the surgical esophageal resection.^13,21–23^ Nearly one-third of the patients who undergo neoadjuvant chemoradiotherapy followed by surgery have a pathologically complete response.^1^ Currently, the need for standard surgical resection in these patients is a topic of debate.^31–34^ Although the advantages of avoiding unnecessary surgery seem clear, medical decisions on surgical versus conservative treatments are often complex. In addition, active surveillance as alternative for standard surgery can be a source of uncertainty and anxiety for patients due to the risk of postponed esophagectomy and the invasiveness of diagnostics used during active surveillance.^35–37^ This complex balance of pros and cons of each strategy emphasizes the need for shared decision-making. Further research on predictive patient and tumor characteristics in patients with a clinically complete response after neoadjuvant chemoradiotherapy is needed to aid patients in the decision process between active surveillance and standard surgery.

This is the first study to assess the association of clinicopathological characteristics with HRQOL over a 6-year postoperative period in patients with locally advanced esophageal cancer who underwent neoadjuvant chemoradiotherapy followed by surgery. Data used for this study were available from a prospective multicenter trial, which resulted in a systematic reporting of all clinicopathological characteristics. Furthermore, this is the first study to assess the relationship between a patient-reported outcome (baseline global HRQOL) and postoperative HRQOL domains for esophageal cancer. All questionnaires used have been well validated.^38,39^

A limitation of this study is that not all eligible patients returned the questionnaire. Although approximately 70% of patients responded, which seems reasonable,^40^ missing questionnaires could possibly impact the results of the present study. Furthermore, in some subgroups the sample sizes were small, especially in the analysis for long-term results, which could have caused a lack of power to detect subtle but potentially relevant differences. Finally, due to the high variability in HRQOL scores over time at the individual level, it is difficult to assess the individual outcomes on the basis of these aggregate analyses.

In conclusion, patients with high baseline global HRQOL or a stage 2 tumor suffer from a more severe deterioration in eating problems, global HRQOL, and fatigue after neoadjuvant chemoradiotherapy plus surgery compared with patients with low baseline global HRQOL or a stage 3 tumor, respectively. The results indicate that patients with locally advanced esophageal cancer in favorable condition at baseline decline more from neoadjuvant chemoradiotherapy and esophagectomy in terms of various quality of life outcomes.

## Contribution to Knowledge

### What does this Study add to Existing Knowledge?

Patients desire detailed information on the expected impact of treatment on their postoperative health-related quality of life. Previously, studies showed that baseline characteristics could be predictive for worse quality of life after primary surgery. The current treatment for esophageal cancer is neoadjuvant therapy followed by esophagectomy. The present study reports that patients in favorable condition seem to decline more in terms of various health-related quality of life endpoints after neoadjuvant chemoradiotherapy followed by esophagectomy.

### What are the Key Implications for Public Health Interventions, Practice, or Policy?

The deterioration in quality of life is most possibly due to the surgical resection of the esophagus. After neoadjuvant chemoradiotherapy, nearly one-third of patients have a pathologically complete response. These patients could possibly benefit from the organ-sparing active surveillance strategy. Our study helps to identify patients who suffer most from esophagectomy. This information could help patients to be informed better, and thus able to make a better-informed decision concerning treatment of their esophageal cancer, which possibly includes an organ-sparing treatment in the future.

### Supplementary Information

Below is the link to the electronic supplementary material.

Supplementary file1 (DOCX 559 KB)
