# Supplementary material for: Characteristics Predicting Short-Term and Long-Term Health-Related Quality of Life in Patients with Esophageal Cancer After Neoadjuvant Chemoradiotherapy and Esophagectomy
Source: Ann Surg Oncol. 2023 Aug 16;30(13):8192–202. doi: 10.1245/s10434-023-14028-8 (PMC10625935; doi:10.1245/s10434-023-14028-8)
Supplement: Supplementary file 1 — Supplementary file1 (DOCX 559 KB) [file 10434_2023_14028_MOESM1_ESM.docx]

**Supplementary figure 1 –** global HRQOL of all subgroups

**
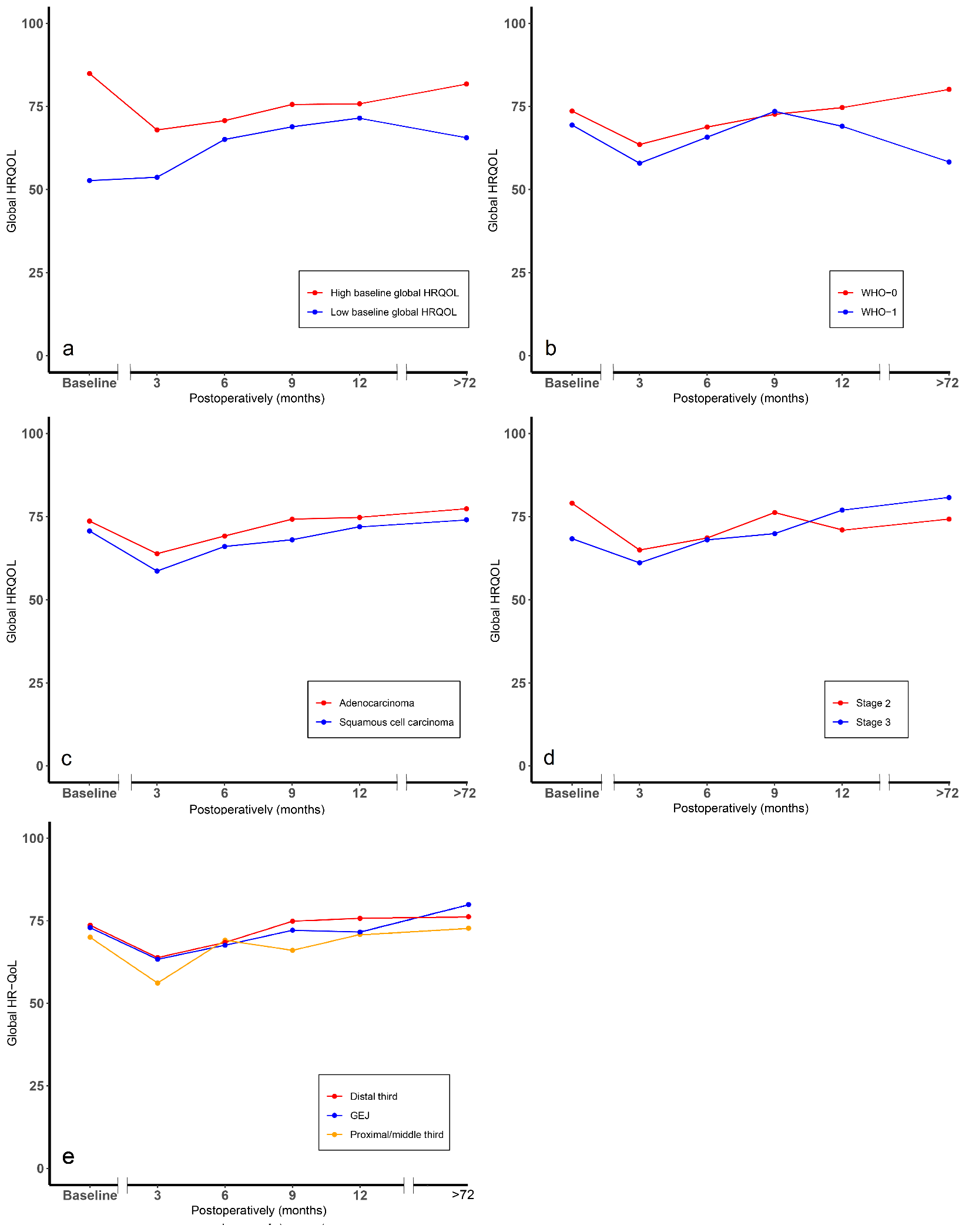
**

**Supplementary Figure 1 -** Mean EORTC-scores representing the global HRQL for patients with **a)** high baseline global HRQOL (red line) or low baseline global HRQOL (blue line) **b)** WHO-0 (red line) or WHO-1 (blue line) **c)** adenocarcinoma (red line) or squamous cell carcinoma (blue line) **d)** stage 2 tumors (red line) or stage 3 tumors (blue line) **e)** tumors located in the proximal/middle third (orange line), tumors in distal third of the esophagus (red line), or tumors at the GEJ (blue line).

Patients with high baseline global HRQOL had a significantly more severe deterioration in short-term and long-term global HRQOL compared to patients with low baseline global HRQOL. The same is true for patients with stage 2 tumor compared to patients with stage 3 tumor. See also supplementary Table 1.

**Supplementary Figure 2** – Fatigue of all subgroups


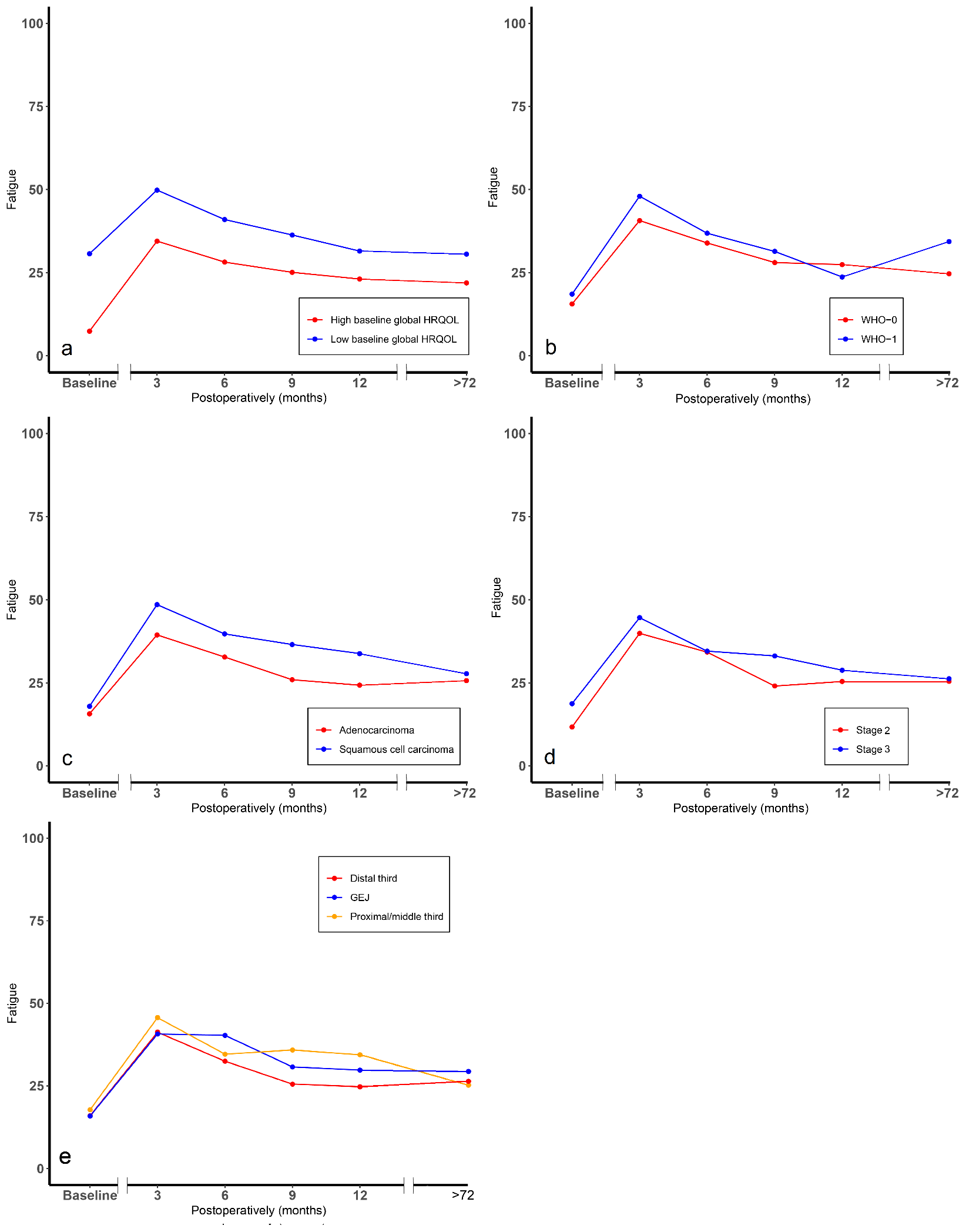


**Supplementary Figure 2 -** Mean EORTC-scores representing fatigue for patients with **a)** high baseline global HRQOL (red line) or low baseline global HRQOL (blue line) **b)** WHO-0 (red line) or WHO-1 (blue line) **c)** adenocarcinoma (red line) or squamous cell carcinoma (blue line) **d)** stage 2 tumors (red line) or stage 3 tumors (blue line) **e)** tumors located in the proximal/middle third (orange line), tumors in distal third of the esophagus (red line), or tumors at the GEJ (blue line).

Patients with high baseline global HRQOL had a clinically relevant more severe deterioration in short-term and long-term fatigue compared to patients with low baseline global HRQOL. See also supplementary Table 2.

**Supplementary Figure 3 –** Emotional problems of all subgroups

**
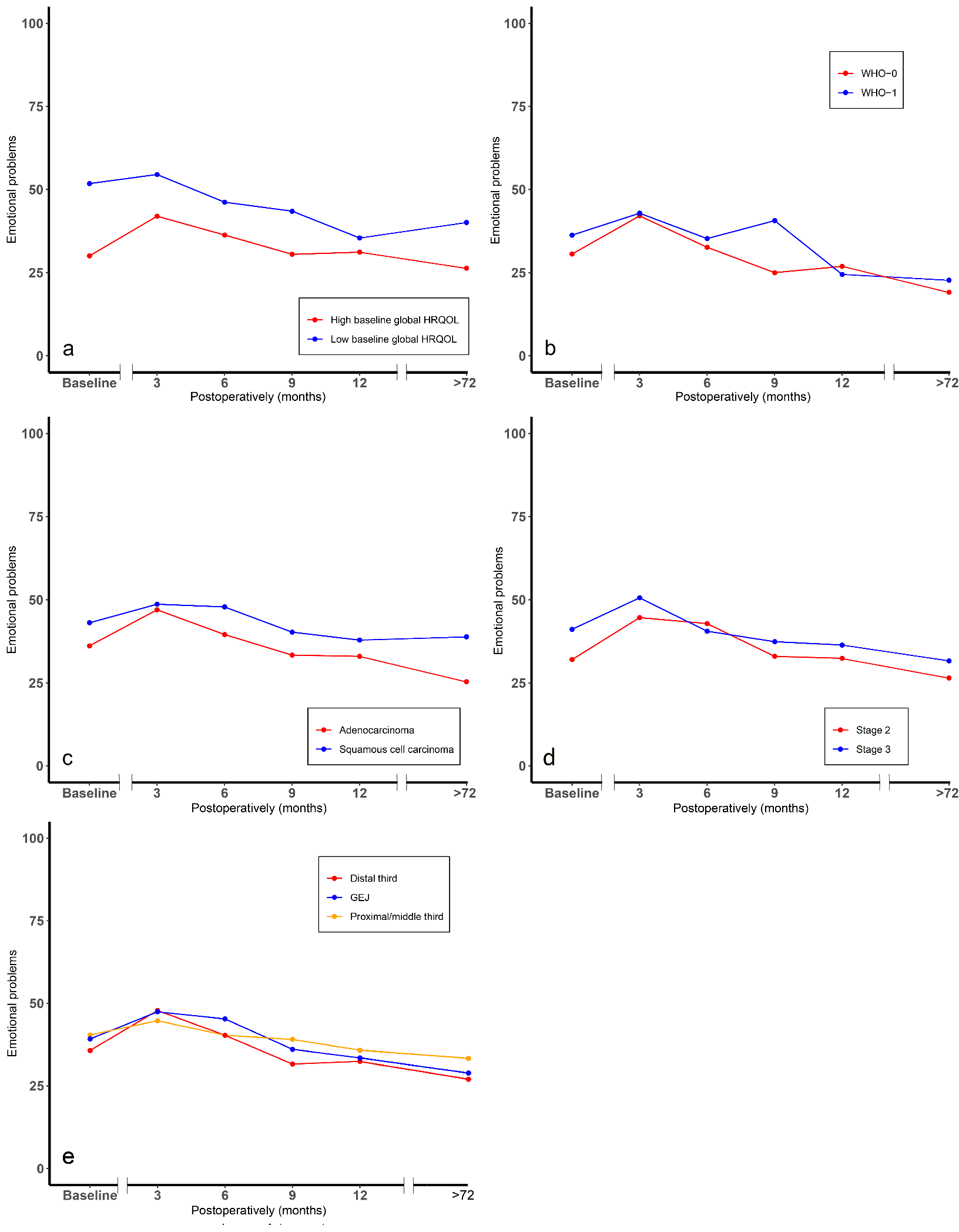
**

**Supplementary Figure 3 -** Mean EORTC-scores representing the emotional problems for patients with **a)** high baseline global HRQOL (red line) or low baseline global HRQOL (blue line) **b)** WHO-0 (red line) or WHO-1 (blue line) **c)** adenocarcinoma (red line) or squamous cell carcinoma (blue line) **d)** stage 2 tumors (red line) or stage 3 tumors (blue line) **e)** tumors located in the proximal/middle third (orange line), tumors in distal third of the esophagus (red line), or tumors at the GEJ (blue line).

No differences were seen between subgroups. See also supplementary Table 3.

**Supplementary Table 1 – overview of differences in improvement or deterioration with respect to global HRQOL between subgroups**

|  | Postoperative timepoint (months) | | | | | | | | | |
| --- | --- | --- | --- | --- | --- | --- | --- | --- | --- | --- |
|  | **3** | | **6** | | **9** | | **12** | | **>72** | |
|  | Δ | P | Δ | p | Δ | p | Δ | p | Δ | p |
| **Baseline gHRQOL** |  |  |  |  |  |  |  |  |  |  |
| Low* vs High | -16 | <0.01 | -26.6 | <0.01 | -25.5 | <0.01 | -28 | <0.01 | -16.1 | 0.02 |
| **WHO** |  |  |  |  |  |  |  |  |  |  |
| 0* vs 1 | -1.4 | 0.24 | 1.2 | 0.51 | 5.1 | 0.68 | -1.4 | 0.37 | 17.7 | 0.09 |
| **Histology** |  |  |  |  |  |  |  |  |  |  |
| AC* vs SCC | -2.3 | 0.70 | -0.2 | 0.93 | -3.2 | 0.86 | 0.2 | 0.91 | 0.3 | 0.40 |
| **Tumor stage** |  |  |  |  |  |  |  |  |  |  |
| 2* vs 3 | 6.8 | 0.46 | 10.1 | 0.30 | 4 | 0.38 | 16.6 | 0.02 | 17.1 | 0.03 |
| **Tumor location** |  |  |  |  |  |  |  |  |  |  |
| Distal* vs prox/mid | 4.1 | 0.96 | -4.3 | 0.87 | 5.0 | 0.51 | 1.3 | 0.77 | -0.1 | 0.56 |
| GEJ* vs prox/mid | 4.3 | 0.96 | -4.5 | 0.55 | 2.9 | 0.61 | -2.2 | 0.70 | 4.3 | 0.81 |
| GEJ* vs distal | 0.2 | 1.00 | -0.1 | 0.25 | -2.0 | 0.74 | -3.4 | 0.36 | 4.4 | 0.33 |
| Δ: Difference between subgroups in improvement or deterioration compared to baseline. A negative value represents a deterioration and a positive value represents an improvement with respect to global HRQOL compared to the reference subgroup.  *Reference subgroup  AC: adenocarcinoma, GEJ: gastroesophageal junction, gHRQOL: global Health Related Quality Of Life, SCC: squamous cell carcinoma, vs: versus, WHO: World Health Organization performance status | | | | | | | | | | |

**Supplementary Table 2 – overview of differences in improvement or deterioration with respect to fatigue between subgroups**

|  | Postoperative timepoint (months) | | | | | | | | | |  |
| --- | --- | --- | --- | --- | --- | --- | --- | --- | --- | --- | --- |
|  | **3** | | **6** | | **9** | | **12** | | **>72** | |  |
|  | Δ | P | Δ | p | Δ | p | Δ | p | Δ | p |  |
| **Baseline gHRQOL** |  |  |  |  |  |  |  |  |  |  |  |
| Low* vs High | 7.9 | 0.03 | 10.5 | 0.013 | 12 | <0.01 | 14.9 | 0.010 | 14.6 | 0.066 |  |
| **WHO** |  |  |  |  |  |  |  |  |  |  |  |
| 0* vs 1 | 4.4 | 0.26 | 0 | 0.47 | 0.4 | 0.23 | -6.7 | 0.62 | 6.8 | 0.38 |  |
| **Histology** |  |  |  |  |  |  |  |  |  |  |  |
| AC* vs SCC | 6.9 | 0.33 | 4.7 | 0.52 | 8.4 | 0.28 | 7.3 | 0.12 | -0.2 | 0.97 |  |
| **Tumor stage** |  |  |  |  |  |  |  |  |  |  |  |
| 2* vs 3 | -2.3 | 0.84 | -6.7 | 0.62 | 2 | 0.24 | -3.6 | 0.93 | -6.3 | 0.11 |  |
| **Tumor location** | |  |  |  |  |  |  |  |  |  |  |
| Distal* vs prox/mid | | -2.6 | 0.97 | -0.3 | 0.69 | -8.5 | 0.62 | -7.9 | 0.67 | 3.0 | 0.24 |
| GEJ* vs prox/mid | | -3.0 | 0.33 | 7.6 | 0.07 | -3.2 | 0.68 | -2.7 | 0.64 | 6.1 | 0.37 |
| GEJ* vs distal | | -0.4 | 0.19 | 7.9 | 0.02 | 5.3 | 0.08 | 5.1 | 0.13 | 3.1 | 0.94 |
| Δ: Difference between subgroups in improvement or deterioration compared to baseline. A negative value represents an improvement and a positive value represents a deterioration with respect to fatigue compared to the reference subgroup.  *Reference subgroup  AC: adenocarcinoma, GEJ: gastroesophageal junction, gHRQOL: global Health Related Quality Of Life, SCC: squamous cell carcinoma, vs: versus, WHO: World Health Organization performance status | | | | | | | | | | |  |

**Supplementary Table 3 – overview of differences in improvement or deterioration with respect to emotional problems between subgroups**

|  | Postoperative time point (months) | | | | | | | | | |  |
| --- | --- | --- | --- | --- | --- | --- | --- | --- | --- | --- | --- |
|  | **3** | | **6** | | **9** | | **12** | | **>72** | |  |
|  | Δ | p | Δ | p | Δ | p | Δ | p | Δ | p |  |
| **Baseline gHRQOL** |  |  |  |  |  |  |  |  |  |  |  |
| Low* vs High | 9.2 | 0.27 | 11.9 | 0.04 | 8.8 | 0.26 | 17.5 | 0.03 | 8 | 0.52 |  |
| **WHO** |  |  |  |  |  |  |  |  |  |  |  |
| 0* vs 1 | 1.6 | 0.28 | -0.2 | 0.12 | -1.6 | 0.39 | -3.8 | 0.45 | 3.7 | 0.27 |  |
| **Histology** |  |  |  |  |  |  |  |  |  |  |  |
| AC* vs SCC | -5.3 | 0.06 | 1.4 | 0.88 | 0 | 0.41 | -2.1 | 0.97 | 6.6 | 0.66 |  |
| **Tumor stage** |  |  |  |  |  |  |  |  |  |  |  |
| 2* vs 3 | -3.2 | 0.74 | -11.4 | 0.16 | -4.7 | 0.74 | -5.1 | 0.53 | -4 | 0.59 |  |
| **Tumor location** | |  |  |  |  |  |  |  |  |  |  |
| Distal* vs prox/mid | | 7.8 | 0.63 | 4.7 | 0.53 | -2.8 | 0.52 | 1.3 | 0.39 | -1.6 | 0.53 |
| GEJ* vs prox/mid | | 3.9 | 0.85 | 6.1 | 0.70 | -1.8 | 0.28 | -1.2 | 0.19 | -3.2 | 0.18 |
| GEJ* vs distal | | -3.9 | 0.39 | 1.5 | 0.74 | 1.0 | 0.38 | -2.5 | 0.30 | -1.6 | 0.31 |
| Δ: Difference between subgroups in improvement or deterioration compared to baseline. A negative value represents an improvement and a positive value represents a deterioration with respect to emotional problems compared to the reference subgroup.  *Reference subgroup  AC: adenocarcinoma, GEJ: gastroesophageal junction, gHRQOL: global Health Related Quality Of Life, SCC: squamous cell carcinoma, vs: versus, WHO: World Health Organization performance status | | | | | | | | | | |  |
